# 4-(1-Ethyl-1*H*-1,3-benzimidazol-2-yl)-*N*,*N*-diphenyl­aniline monohydrate

**DOI:** 10.1107/S160053681100119X

**Published:** 2011-01-15

**Authors:** Tao Wu, Kai Wang, Peng Jiang, Hong-Jun Zhu

**Affiliations:** a Department of Applied Chemistry, College of Science, Nanjing University of Technology, Nanjing 210009, People’s Republic of China

## Abstract

In the title compound, C_27_H_23_N_3_O·H_2_O, the benzimidazole ring system has an r.m.s. deviation of 0.0071 Å and makes dihedral angles of 34.51 (2), 55.22 (3) and 41.05 (5)° with the central and N-bonded phenyl rings, respectively. In the crystal, the water mol­ecular is connected to the organic mol­ecule by inter­molecular O—H⋯N hydrogen bonds. Weak inter­molecular C—H⋯O hydrogen bonds also occur.

## Related literature

For the synthetic procedure, see: Vinodkumar *et al.* (2008 ▶). For bond-length data, see: Allen *et al.* (1987 ▶). For background to the use of the title compound as an inter­mediate in the preparation of OLED materials, see: Kakimoto *et al.* (2008 ▶). 
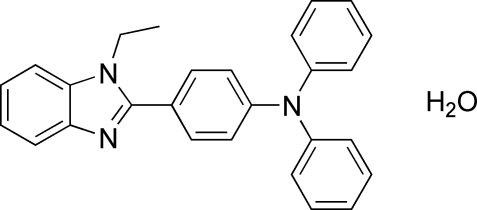


## Experimental

### 

#### Crystal data

C_27_H_23_N_3_·H_2_O
*M* *_r_* = 407.50
Monoclinic, 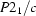

*a* = 12.278 (3) Å
*b* = 9.2690 (19) Å
*c* = 19.468 (4) Å
β = 97.81 (3)°
*V* = 2195.0 (8) Å^3^
*Z* = 4
Mo *K*α radiation
μ = 0.08 mm^−1^
*T* = 293 K
0.30 × 0.20 × 0.10 mm

#### Data collection

Enraf–Nonius CAD-4 diffractometer
Absorption correction: ψ scan (North *et al.*, 1968 ▶) *T*_min_ = 0.978, *T*_max_ = 0.992
4223 measured reflections
4024 independent reflections
2487 reflections with *I* > 2σ(*I*)
*R*_int_ = 0.025
3 standard reflections every 200 reflections  intensity decay: 1%

#### Refinement

*R*[*F* ^2^ > 2σ(*F* ^2^)] = 0.057
*wR*(*F* ^2^) = 0.169
*S* = 1.01
4024 reflections
281 parameters
H-atom parameters constrained
Δρ_max_ = 0.18 e Å^−3^
Δρ_min_ = −0.26 e Å^−3^

### 

Data collection: *CAD-4 Software* (Enraf–Nonius, 1985 ▶); cell refinement: *CAD-4 Software*; data reduction: *XCAD4* (Harms & Wocadlo, 1995 ▶); program(s) used to solve structure: *SHELXS97* (Sheldrick, 2008 ▶); program(s) used to refine structure: *SHELXL97* (Sheldrick, 2008 ▶); molecular graphics: *SHELXTL* (Sheldrick, 2008 ▶); software used to prepare material for publication: *SHELXTL*.

## Supplementary Material

Crystal structure: contains datablocks I, global. DOI: 10.1107/S160053681100119X/bq2268sup1.cif

Structure factors: contains datablocks I. DOI: 10.1107/S160053681100119X/bq2268Isup2.hkl

Additional supplementary materials:  crystallographic information; 3D view; checkCIF report

## Figures and Tables

**Table 1:** Hydrogen-bond geometry (Å, °)

*D*—H⋯*A*	*D*—H	H⋯*A*	*D*⋯*A*	*D*—H⋯*A*
O*W*—H*WB*⋯N2	0.85	2.50	2.903 (3)	110
O*W*—H*WA*⋯N2	0.85	2.49	2.903 (3)	111
C24—H24*A*⋯O*W*^i^	0.93	2.43	3.352 (4)	173

Symmetry code: (i) 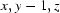.

**Fig. 1.**
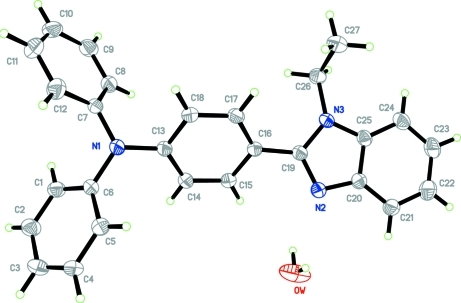
The molecular structure of (I), with the atom-numbering scheme. Displacement ellipsoids are drawn at the 50% probability level.

**Fig. 2.**
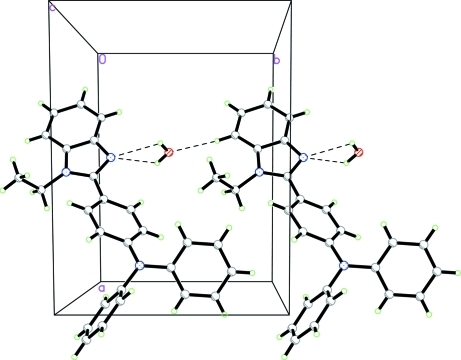
A packing diagram of (I). Hydrogen bonds are shown as dashed lines.

## supplementary crystallographic information

### Comment

The title compound, (I), is a kind of important organic intermediate which can be used for many fields such as OLED materials. (Kakimoto *et al.*, 2008). We herein report its crystal structure.

In the molecule of (I), (Fig.1), the bond lengths (Allen *et al.*, 1987) and angles are within normal ranges. The benzimidazole ring (A) is obviously almost coplanar with an r.m.s. deviation of 0.0071 °. The dihedral angles between A and the rest three phenyl rings B (C13-C18), C (C7-C12) and D (C1-C6) are 34.51 (2) ° 55.22 (3) ° and 41.05 (5) °, respectively. The H_2_O molecule stems from the solvent ethanol, and the water molecular is connected with the target molecular by intermolecular C—H···O and O—H···N hydrogen bonds (Table 1), which seems to be very effective in the stabilization of the crystal structure.

### Experimental

The title compound, (I) was synthesized according to the literature (Vinodkumar *et al.*, 2008) The crystals were obtained by dissolving (I) (0.52 g, 1.28 mmol) in 25 ml ethanol and evaporating the solvent slowly at room temperature for about 7 d.

### Refinement

H atoms bonded to N and O atoms were located in a difference map and refined with distance restraints of O—H = 0.85 (2) and N—H = 0.90 (2) Å, and with *U*_iso_(H) = *1.2U*_eq_(N,*O*). Other H atoms were positioned geometrically and refined using a riding model, with C—H = 0.93 - 0.97 (2) Å, *U*_iso_(H) = *1.2U*_eq_(C).

### Crystal data

**Table:**

C_27_H_23_N_3_·H_2_O	*F*(000) = 864
*M**_r_* = 407.50	*D*_x_ = 1.233 Mg m^−^^3^
Monoclinic, *P*2_1_/*c*	Mo *K*α radiation, λ = 0.71073 Å
Hall symbol: -P 2ybc	Cell parameters from 25 reflections
*a* = 12.278 (3) Å	θ = 10–14°
*b* = 9.2690 (19) Å	µ = 0.08 mm^−^^1^
*c* = 19.468 (4) Å	*T* = 293 K
β = 97.81 (3)°	Block, colorless
*V* = 2195.0 (8) Å^3^	0.30 × 0.20 × 0.10 mm
*Z* = 4	

### Data collection

**Table:**

Enraf–Nonius CAD-4 diffractometer	2487 reflections with *I* > 2σ(*I*)
Radiation source: fine-focus sealed tube	*R*_int_ = 0.025
graphite	θ_max_ = 25.4°, θ_min_ = 1.7°
ω/2θ scans	*h* = 0→14
Absorption correction: ψ scan (North *et al.*, 1968)	*k* = 0→11
*T*_min_ = 0.978, *T*_max_ = 0.992	*l* = −23→23
4223 measured reflections	3 standard reflections every 200 reflections
4024 independent reflections	intensity decay: 1%

### Refinement

**Table:**

Refinement on *F*^2^	Secondary atom site location: difference Fourier map
Least-squares matrix: full	Hydrogen site location: inferred from neighbouring sites
*R*[*F*^2^ > 2σ(*F*^2^)] = 0.057	H-atom parameters constrained
*wR*(*F*^2^) = 0.169	*w* = 1/[σ^2^(*F*_o_^2^) + (0.090*P*)^2^] where *P* = (*F*_o_^2^ + 2*F*_c_^2^)/3
*S* = 1.01	(Δ/σ)_max_ < 0.001
4024 reflections	Δρ_max_ = 0.18 e Å^−^^3^
281 parameters	Δρ_min_ = −0.26 e Å^−^^3^
0 restraints	Extinction correction: *SHELXL97* (Sheldrick, 2008), Fc^*^=kFc[1+0.001xFc^2^λ^3^/sin(2θ)]^-1/4^
Primary atom site location: structure-invariant direct methods	Extinction coefficient: 0.0093 (17)

### Special details

**Table:**

Geometry. All e.s.d.'s (except the e.s.d. in the dihedral angle between two l.s. planes) are estimated using the full covariance matrix. The cell e.s.d.'s are taken into account individually in the estimation of e.s.d.'s in distances, angles and torsion angles; correlations between e.s.d.'s in cell parameters are only used when they are defined by crystal symmetry. An approximate (isotropic) treatment of cell e.s.d.'s is used for estimating e.s.d.'s involving l.s. planes.
Refinement. Refinement of *F*^2^ against ALL reflections. The weighted *R*-factor *wR* and goodness of fit *S* are based on *F*^2^, conventional *R*-factors *R* are based on *F*, with *F* set to zero for negative *F*^2^. The threshold expression of *F*^2^ > σ(*F*^2^) is used only for calculating *R*-factors(gt) *etc*. and is not relevant to the choice of reflections for refinement. *R*-factors based on *F*^2^ are statistically about twice as large as those based on *F*, and *R*- factors based on ALL data will be even larger.

### Fractional atomic coordinates and isotropic or equivalent isotropic displacement parameters (Å^2^)

**Table:**

	*x*	*y*	*z*	*U*_iso_*/*U*_eq_	
N1	0.12417 (17)	0.8084 (2)	−0.08205 (12)	0.0535 (6)	
C1	0.0167 (2)	1.0299 (3)	−0.09280 (14)	0.0502 (7)	
H1A	−0.0468	0.9764	−0.0906	0.060*	
N2	0.53509 (16)	0.6320 (2)	0.14215 (10)	0.0457 (5)	
C2	0.0102 (2)	1.1782 (3)	−0.10031 (16)	0.0599 (8)	
H2B	−0.0578	1.2238	−0.1038	0.072*	
N3	0.47737 (16)	0.4037 (2)	0.12179 (11)	0.0435 (5)	
C3	0.1033 (3)	1.2582 (3)	−0.10260 (16)	0.0622 (8)	
H3A	0.0986	1.3580	−0.1072	0.075*	
C4	0.2031 (2)	1.1914 (3)	−0.09818 (15)	0.0567 (7)	
H4A	0.2664	1.2461	−0.0993	0.068*	
C5	0.2108 (2)	1.0429 (3)	−0.09211 (14)	0.0522 (7)	
H5A	0.2787	0.9977	−0.0905	0.063*	
C6	0.1169 (2)	0.9614 (3)	−0.08850 (13)	0.0442 (6)	
C7	0.03574 (19)	0.7225 (3)	−0.11420 (13)	0.0419 (6)	
C8	−0.0220 (2)	0.6349 (3)	−0.07465 (15)	0.0514 (7)	
H8A	−0.0017	0.6299	−0.0269	0.062*	
C9	−0.1095 (2)	0.5549 (3)	−0.10554 (18)	0.0625 (8)	
H9A	−0.1477	0.4955	−0.0786	0.075*	
C10	−0.1405 (3)	0.5620 (3)	−0.1750 (2)	0.0737 (10)	
H10A	−0.2004	0.5088	−0.1956	0.088*	
C11	−0.0834 (3)	0.6476 (4)	−0.21467 (17)	0.0733 (9)	
H11A	−0.1042	0.6516	−0.2624	0.088*	
C12	0.0048 (2)	0.7282 (3)	−0.18469 (14)	0.0609 (8)	
H12A	0.0433	0.7861	−0.2121	0.073*	
C13	0.20766 (19)	0.7440 (3)	−0.03508 (13)	0.0431 (6)	
C14	0.2497 (2)	0.8104 (3)	0.02666 (13)	0.0461 (6)	
H14A	0.2234	0.9005	0.0375	0.055*	
C15	0.3298 (2)	0.7442 (3)	0.07189 (13)	0.0443 (6)	
H15A	0.3578	0.7910	0.1128	0.053*	
C16	0.37033 (19)	0.6077 (3)	0.05776 (13)	0.0408 (6)	
C17	0.32912 (19)	0.5432 (3)	−0.00436 (13)	0.0435 (6)	
H17A	0.3560	0.4536	−0.0154	0.052*	
C18	0.2488 (2)	0.6091 (3)	−0.05033 (13)	0.0446 (6)	
H18A	0.2220	0.5633	−0.0917	0.053*	
C19	0.4598 (2)	0.5481 (3)	0.10699 (13)	0.0419 (6)	
C20	0.6054 (2)	0.5389 (3)	0.18193 (13)	0.0457 (6)	
C21	0.6991 (2)	0.5681 (3)	0.22815 (14)	0.0560 (7)	
H21A	0.7225	0.6625	0.2371	0.067*	
C22	0.7562 (3)	0.4543 (4)	0.26021 (16)	0.0706 (9)	
H22A	0.8196	0.4716	0.2910	0.085*	
C23	0.7204 (3)	0.3122 (3)	0.24720 (16)	0.0681 (9)	
H23A	0.7611	0.2374	0.2697	0.082*	
C24	0.6278 (2)	0.2793 (3)	0.20264 (15)	0.0587 (8)	
H24A	0.6040	0.1847	0.1947	0.070*	
C25	0.5710 (2)	0.3962 (3)	0.16973 (13)	0.0443 (6)	
C26	0.4069 (2)	0.2785 (3)	0.10055 (15)	0.0556 (7)	
H26A	0.3350	0.3125	0.0803	0.067*	
H26B	0.3975	0.2221	0.1413	0.067*	
C27	0.4534 (3)	0.1826 (3)	0.04887 (17)	0.0717 (9)	
H27A	0.4046	0.1029	0.0370	0.108*	
H27B	0.5241	0.1471	0.0689	0.108*	
H27C	0.4611	0.2371	0.0079	0.108*	
OW	0.5578 (2)	0.9300 (3)	0.18768 (15)	0.1158 (10)	
HWB	0.5151	0.8673	0.2015	0.139*	
HWA	0.5919	0.8888	0.1579	0.139*	

### Atomic displacement parameters (Å^2^)

**Table:**

	*U*^11^	*U*^22^	*U*^33^	*U*^12^	*U*^13^	*U*^23^
N1	0.0452 (12)	0.0373 (12)	0.0720 (15)	−0.0054 (10)	−0.0136 (11)	0.0103 (11)
C1	0.0447 (15)	0.0436 (16)	0.0618 (18)	−0.0048 (12)	0.0060 (13)	−0.0013 (13)
N2	0.0466 (12)	0.0395 (12)	0.0487 (13)	0.0007 (10)	−0.0017 (10)	0.0037 (10)
C2	0.0593 (18)	0.0438 (17)	0.076 (2)	0.0047 (14)	0.0084 (15)	−0.0064 (14)
N3	0.0456 (12)	0.0314 (11)	0.0524 (13)	−0.0009 (9)	0.0030 (10)	0.0064 (9)
C3	0.076 (2)	0.0335 (15)	0.076 (2)	−0.0049 (15)	0.0059 (17)	−0.0042 (14)
C4	0.0601 (18)	0.0439 (17)	0.0637 (18)	−0.0169 (14)	−0.0005 (14)	0.0051 (14)
C5	0.0432 (14)	0.0482 (16)	0.0631 (18)	−0.0050 (13)	−0.0007 (13)	0.0064 (14)
C6	0.0455 (14)	0.0373 (14)	0.0476 (15)	−0.0025 (12)	−0.0012 (12)	0.0054 (12)
C7	0.0381 (13)	0.0333 (13)	0.0520 (16)	−0.0002 (11)	−0.0019 (12)	0.0013 (11)
C8	0.0511 (15)	0.0469 (16)	0.0554 (17)	−0.0007 (13)	0.0041 (13)	0.0050 (13)
C9	0.0478 (16)	0.0487 (17)	0.090 (2)	−0.0096 (14)	0.0067 (16)	0.0052 (16)
C10	0.0583 (19)	0.0508 (19)	0.104 (3)	−0.0061 (16)	−0.0186 (19)	−0.0158 (19)
C11	0.085 (2)	0.071 (2)	0.0557 (19)	0.004 (2)	−0.0171 (18)	−0.0122 (17)
C12	0.0669 (19)	0.0648 (19)	0.0495 (17)	−0.0021 (16)	0.0026 (15)	0.0044 (14)
C13	0.0378 (13)	0.0357 (14)	0.0544 (16)	−0.0037 (11)	0.0013 (12)	0.0076 (12)
C14	0.0448 (14)	0.0386 (14)	0.0537 (16)	0.0036 (12)	0.0025 (12)	−0.0012 (12)
C15	0.0458 (14)	0.0399 (14)	0.0458 (15)	−0.0016 (12)	0.0009 (12)	−0.0031 (11)
C16	0.0377 (13)	0.0367 (14)	0.0476 (15)	−0.0032 (11)	0.0040 (11)	0.0025 (11)
C17	0.0415 (13)	0.0332 (13)	0.0560 (16)	−0.0002 (11)	0.0073 (12)	−0.0011 (12)
C18	0.0450 (14)	0.0381 (14)	0.0487 (15)	−0.0049 (12)	−0.0003 (12)	−0.0016 (12)
C19	0.0430 (13)	0.0360 (14)	0.0469 (14)	−0.0002 (12)	0.0063 (11)	0.0028 (12)
C20	0.0463 (15)	0.0440 (15)	0.0460 (15)	0.0011 (12)	0.0041 (12)	0.0036 (12)
C21	0.0570 (17)	0.0506 (17)	0.0563 (17)	−0.0022 (14)	−0.0067 (14)	0.0013 (14)
C22	0.0651 (19)	0.076 (2)	0.064 (2)	0.0051 (18)	−0.0142 (16)	0.0095 (17)
C23	0.072 (2)	0.061 (2)	0.067 (2)	0.0176 (17)	−0.0079 (17)	0.0206 (16)
C24	0.0676 (19)	0.0433 (16)	0.0645 (19)	0.0062 (14)	0.0067 (16)	0.0122 (14)
C25	0.0469 (14)	0.0404 (14)	0.0458 (15)	0.0033 (12)	0.0072 (12)	0.0076 (12)
C26	0.0544 (16)	0.0392 (15)	0.0715 (19)	−0.0095 (13)	0.0032 (14)	0.0105 (14)
C27	0.077 (2)	0.0437 (17)	0.091 (2)	−0.0017 (16)	−0.0020 (18)	−0.0060 (16)
OW	0.154 (2)	0.0616 (15)	0.146 (2)	−0.0265 (16)	0.073 (2)	−0.0184 (15)

### Geometric parameters (Å, °)

**Table:**

N1—C13	1.410 (3)	C13—C14	1.386 (4)
N1—C7	1.421 (3)	C13—C18	1.396 (3)
N1—C6	1.426 (3)	C14—C15	1.373 (3)
C1—C6	1.377 (3)	C14—H14A	0.9300
C1—C2	1.383 (4)	C15—C16	1.401 (3)
C1—H1A	0.9300	C15—H15A	0.9300
N2—C19	1.324 (3)	C16—C17	1.382 (3)
N2—C20	1.381 (3)	C16—C19	1.464 (3)
C2—C3	1.369 (4)	C17—C18	1.380 (3)
C2—H2B	0.9300	C17—H17A	0.9300
N3—C19	1.380 (3)	C18—H18A	0.9300
N3—C25	1.381 (3)	C20—C21	1.388 (3)
N3—C26	1.473 (3)	C20—C25	1.398 (4)
C3—C4	1.365 (4)	C21—C22	1.369 (4)
C3—H3A	0.9300	C21—H21A	0.9300
C4—C5	1.384 (4)	C22—C23	1.401 (4)
C4—H4A	0.9300	C22—H22A	0.9300
C5—C6	1.388 (3)	C23—C24	1.368 (4)
C5—H5A	0.9300	C23—H23A	0.9300
C7—C12	1.374 (4)	C24—C25	1.397 (3)
C7—C8	1.380 (3)	C24—H24A	0.9300
C8—C9	1.375 (4)	C26—C27	1.511 (4)
C8—H8A	0.9300	C26—H26A	0.9700
C9—C10	1.356 (5)	C26—H26B	0.9700
C9—H9A	0.9300	C27—H27A	0.9600
C10—C11	1.365 (5)	C27—H27B	0.9600
C10—H10A	0.9300	C27—H27C	0.9600
C11—C12	1.378 (4)	OW—HWB	0.8499
C11—H11A	0.9300	OW—HWA	0.8501
C12—H12A	0.9300		
			
C13—N1—C7	119.5 (2)	C13—C14—H14A	119.7
C13—N1—C6	120.7 (2)	C14—C15—C16	121.4 (2)
C7—N1—C6	118.9 (2)	C14—C15—H15A	119.3
C6—C1—C2	120.2 (3)	C16—C15—H15A	119.3
C6—C1—H1A	119.9	C17—C16—C15	117.7 (2)
C2—C1—H1A	119.9	C17—C16—C19	124.3 (2)
C19—N2—C20	105.2 (2)	C15—C16—C19	117.8 (2)
C3—C2—C1	120.4 (3)	C18—C17—C16	121.4 (2)
C3—C2—H2B	119.8	C18—C17—H17A	119.3
C1—C2—H2B	119.8	C16—C17—H17A	119.3
C19—N3—C25	106.37 (19)	C17—C18—C13	120.4 (2)
C19—N3—C26	129.6 (2)	C17—C18—H18A	119.8
C25—N3—C26	123.6 (2)	C13—C18—H18A	119.8
C4—C3—C2	119.9 (3)	N2—C19—N3	112.5 (2)
C4—C3—H3A	120.0	N2—C19—C16	121.7 (2)
C2—C3—H3A	120.0	N3—C19—C16	125.7 (2)
C3—C4—C5	120.4 (3)	N2—C20—C21	129.9 (2)
C3—C4—H4A	119.8	N2—C20—C25	110.1 (2)
C5—C4—H4A	119.8	C21—C20—C25	120.0 (2)
C4—C5—C6	119.9 (3)	C22—C21—C20	118.2 (3)
C4—C5—H5A	120.1	C22—C21—H21A	120.9
C6—C5—H5A	120.1	C20—C21—H21A	120.9
C1—C6—C5	119.2 (2)	C21—C22—C23	120.9 (3)
C1—C6—N1	120.6 (2)	C21—C22—H22A	119.6
C5—C6—N1	120.2 (2)	C23—C22—H22A	119.6
C12—C7—C8	119.1 (2)	C24—C23—C22	122.6 (3)
C12—C7—N1	120.6 (2)	C24—C23—H23A	118.7
C8—C7—N1	120.3 (2)	C22—C23—H23A	118.7
C9—C8—C7	120.3 (3)	C23—C24—C25	116.0 (3)
C9—C8—H8A	119.9	C23—C24—H24A	122.0
C7—C8—H8A	119.9	C25—C24—H24A	122.0
C10—C9—C8	120.4 (3)	N3—C25—C24	131.8 (2)
C10—C9—H9A	119.8	N3—C25—C20	105.8 (2)
C8—C9—H9A	119.8	C24—C25—C20	122.4 (2)
C9—C10—C11	119.8 (3)	N3—C26—C27	112.9 (2)
C9—C10—H10A	120.1	N3—C26—H26A	109.0
C11—C10—H10A	120.1	C27—C26—H26A	109.0
C10—C11—C12	120.6 (3)	N3—C26—H26B	109.0
C10—C11—H11A	119.7	C27—C26—H26B	109.0
C12—C11—H11A	119.7	H26A—C26—H26B	107.8
C7—C12—C11	119.8 (3)	C26—C27—H27A	109.5
C7—C12—H12A	120.1	C26—C27—H27B	109.5
C11—C12—H12A	120.1	H27A—C27—H27B	109.5
C14—C13—C18	118.6 (2)	C26—C27—H27C	109.5
C14—C13—N1	121.8 (2)	H27A—C27—H27C	109.5
C18—C13—N1	119.6 (2)	H27B—C27—H27C	109.5
C15—C14—C13	120.5 (2)	HWB—OW—HWA	106.9
C15—C14—H14A	119.7		
			
C6—C1—C2—C3	−0.9 (4)	C19—C16—C17—C18	−176.2 (2)
C1—C2—C3—C4	0.7 (5)	C16—C17—C18—C13	0.4 (4)
C2—C3—C4—C5	0.7 (4)	C14—C13—C18—C17	0.5 (4)
C3—C4—C5—C6	−1.8 (4)	N1—C13—C18—C17	−178.8 (2)
C2—C1—C6—C5	−0.3 (4)	C20—N2—C19—N3	0.0 (3)
C2—C1—C6—N1	−178.8 (3)	C20—N2—C19—C16	−179.3 (2)
C4—C5—C6—C1	1.6 (4)	C25—N3—C19—N2	−0.2 (3)
C4—C5—C6—N1	−179.8 (3)	C26—N3—C19—N2	172.0 (2)
C13—N1—C6—C1	−136.8 (3)	C25—N3—C19—C16	179.0 (2)
C7—N1—C6—C1	32.2 (4)	C26—N3—C19—C16	−8.7 (4)
C13—N1—C6—C5	44.7 (4)	C17—C16—C19—N2	142.4 (3)
C7—N1—C6—C5	−146.3 (2)	C15—C16—C19—N2	−32.3 (3)
C13—N1—C7—C12	−130.9 (3)	C17—C16—C19—N3	−36.8 (4)
C6—N1—C7—C12	59.9 (3)	C15—C16—C19—N3	148.5 (2)
C13—N1—C7—C8	50.7 (3)	C19—N2—C20—C21	179.5 (3)
C6—N1—C7—C8	−118.5 (3)	C19—N2—C20—C25	0.2 (3)
C12—C7—C8—C9	−0.2 (4)	N2—C20—C21—C22	−178.5 (3)
N1—C7—C8—C9	178.2 (2)	C25—C20—C21—C22	0.6 (4)
C7—C8—C9—C10	−0.5 (4)	C20—C21—C22—C23	−0.6 (5)
C8—C9—C10—C11	1.0 (5)	C21—C22—C23—C24	−0.2 (5)
C9—C10—C11—C12	−0.7 (5)	C22—C23—C24—C25	0.8 (5)
C8—C7—C12—C11	0.5 (4)	C19—N3—C25—C24	−179.4 (3)
N1—C7—C12—C11	−177.9 (3)	C26—N3—C25—C24	7.8 (4)
C10—C11—C12—C7	−0.1 (5)	C19—N3—C25—C20	0.4 (3)
C7—N1—C13—C14	−137.3 (3)	C26—N3—C25—C20	−172.5 (2)
C6—N1—C13—C14	31.7 (4)	C23—C24—C25—N3	178.9 (3)
C7—N1—C13—C18	42.1 (3)	C23—C24—C25—C20	−0.8 (4)
C6—N1—C13—C18	−149.0 (2)	N2—C20—C25—N3	−0.4 (3)
C18—C13—C14—C15	−0.3 (4)	C21—C20—C25—N3	−179.7 (2)
N1—C13—C14—C15	179.0 (2)	N2—C20—C25—C24	179.4 (2)
C13—C14—C15—C16	−0.9 (4)	C21—C20—C25—C24	0.0 (4)
C14—C15—C16—C17	1.8 (4)	C19—N3—C26—C27	110.2 (3)
C14—C15—C16—C19	176.8 (2)	C25—N3—C26—C27	−78.7 (3)
C15—C16—C17—C18	−1.6 (4)		

### Hydrogen-bond geometry (Å, °)

**Table:**

*D*—H···*A*	*D*—H	H···*A*	*D*···*A*	*D*—H···*A*
OW—HWB···N2	0.85	2.50	2.903 (3)	110
OW—HWA···N2	0.85	2.49	2.903 (3)	111
C24—H24A···OW^i^	0.93	2.43	3.352 (4)	173

Symmetry codes: (i) *x*, *y*−1, *z*.
